# Supplementary material for: Topological and enzymatic analysis of human Alg2 mannosyltransferase reveals its role in lipid-linked oligosaccharide biosynthetic pathway
Source: Commun Biol. 2022 Feb 8;5:117. doi: 10.1038/s42003-022-03066-9 (PMC8827073; doi:10.1038/s42003-022-03066-9)
Supplement: Supplementary file 2 — Description of Additional Supplementary Files [file 42003_2022_3066_MOESM2_ESM.pdf]

Description of Additional Supplementary Files

File name: Supplementary Data 1
Description: Source data for the graphs and charts in the figures.
